# Thyroid function in healthy and unhealthy preterm newborns

**DOI:** 10.4314/ahs.v18i2.23

**Published:** 2018-06

**Authors:** Gökten Korkmaz, Mustafa Özçetin, Yakup Çağ, Ufuk Yükselmiş, Volkan Öngel, Olcay Işık

**Affiliations:** 1 Süleymaniye Maternity and Children Diseases Training and Research Hospital, Department of Pediatrics, Istanbul, Turkey; 2 Istanbul University, Istanbul Faculty of Medicine, Department of Pediatrics, Istanbul, Turkey; 3 Dr. Lutfi Kirdar Kartal Training and Research Hospital, Department of Pediatrics, Istanbul, Turkey; 4 Beykent University, Department of Economics, Istanbul, Turkey; 5 Kocaeli University Faculty of Medicine, Department of Neonatology, Kocaeli, Turkey

**Keywords:** Prematurity, hypothyroxinemia, thyroid, TSH

## Abstract

**Background:**

The thyroid gland and hormonal regulation are among the most important systems to be investigated in pre-term infants. This study sought to investigate thyroid hormone levels of healthy and unhealthy pre-term infants.

**Methods:**

The prospective study included 53 consecutive premature infants admitted to the neonatal intensive care unit within a duration of one year. Of these preterm babies, 20 were healthy, while 33 had problems such as asphyxia or RDS. Venous blood samples were collected at baseline 0–24 hours, 7 and 14 days and FT3, FT4, and TSH levels were determined. Other data recorded included demographic characteristics of the patients and clinical variables.

**Results:**

The most frequent health problems were RDS (87.9%), sepsis (30.3%), and retinopathy of prematurity (24.2%). The mean TSH levels showed a consistent decline at three consequent measurements in both groups, which were always significantly lower in unhealthy pre-terms. In both groups, TSH levels showed significant decreases on Day 7 and Day 14 compared to the baseline levels (p<005). The levels of FT3 and FT4 consistently showed significant correlations with gestational week and birth weight at each of the three measurements.

**Conclusion:**

Pre-term infants, especially those having problems, have significant hypothyroxinemia that may require thyroid hormone replacement therapy.

## Introduction

Thyroid hormones are the cornerstones of a complex system that plays an important role in the growth and development of children, especially in the development of the nervous system and brain. Therefore, even minimal disruptions of this system can cause permanent damage. Unrecognized early hypothyroidism resulting in mental retardation is a striking example of this condition.^1^,^2^

Thanks to the technological improvements in neonatal intensive care units, the mortality rate of premature infants has considerably improved, reducing the limit of viability to as early as 24 weeks of gestational age. This reduction in mortality rates of premature babies has drawn attention to increased morbidity issues.^3^ For those infants who have developmental problems in many aspects, improving growth and development to optimum levels is among one of the leading problems to be dealt with.^4^ The thyroid gland and hormonal regulation are among the most important systems to be studied in pre-term infants.^5^

Thyroid dysfunction is a common problem in pre-term infants.^3^,^6^ Hypothyroxinemia of prematurity within the first month of life may represent important prognostic information about morbidity and mortality. Thyroid hormone synthesis may be disrupted in co-morbid conditions (sepsis, RDS, shock, asphyxia, etc.), worsening the metabolism of premature infants and causing higher hormone levels compared to healthy infants.^6^,^7^ This study aimed to investigate thyroid hormone levels of healthy and unhealthy pre-term infants.

## Materials and methods

This prospective study included 53 consecutive premature infants (26 boys, 27 girls) who were admitted to the neonatal intensive care unit of pediatrics department of Okmeydanı Training and Research Hospital, İstanbul, Turkey, between August 2009 and August 2010. Of these preterm babies, 20 were healthy (group 1), while 33 had problems such as asphyxia, respiratory distress syndrome (RDS), or meconium aspiration syndrome (group 2). Infants whose mothers had thyroid disease or use of alcohol, cigarettes or drugs during pregnancy and who died within the first month of life were excluded. Approval for the study was obtained from the Local Ethics Committee of the Okmeydanı Training and Research Hospital (No:254/2009). Written informed consent was obtained from all the parents.

Data were collected on demographic characteristics of the patients, perinatal risk factors, length of hospitalization, survival, the need for surfactant therapy, antibiotic treatment, and mechanical ventilation, and the development of RDS, necrotizing enterocolitis, sepsis, and retinopathy of prematurity. Gestational age was determined as completed gestational weeks after the onset of the last normal menstrual period by obstetric examination, standard obstetric parameters, and the New Ballard Score, which was used especially in cases in which data on maternal and gestational age were not clearly elicited.^8^ Growth retardation at birth was assessed by reference to the growth curves defined by Lubchenco.^9^ Small for gestational age (SGA) was defined as birth weight below the 10^th^ percentile for the gestational age using the Lubchenco's growth curves.^9^

Venous blood samples (3 ml) were collected from all cases into sterile dry glass tubes at baseline (0–24 hours),^7^, and 14 days and centrifuged within an hour. Blood collection coincided with routine morning blood sampling (7 to 9 AM). Levels of free tri-iodothyronine (FT3), free thyroxine (FT4), and thyroid-stimulating hormone (TSH) were determined on a Beckman Coulter DXI 800 auto analyzer using the immunochemiluminescent method.

Data was analyzed using the NCSS (Number Cruncher Statistical System) 2007 and the PASS (Power Analysis and Sample Size) 2008 statistical software (Utah, USA). Continuous variables were expressed as mean and standard deviation, and categorical variables as frequencies. The Student-t test and Mann-Whitney U-test were used to compare the two groups for parameters with and without normal distribution, respectively. For in-group comparisons, normally distributed data were compared using analysis of variance for repeated measures, and the Bonferroni test for the assessment of the day that was associated with a significant result. The corresponding tests for non-normally distributed data were the Friedman test and the Wilcoxon signed-rank test. A P value of less than 0.05 was considered to be significant.

## Results

Data on gender, type of delivery, gestational week, and birth weight of healthy and unhealthy pre-term babies are shown in Table 1. The babies were delivered either by caesarean section (92.5%) or vaginal delivery (7.5%) at a mean of 32.1±3.2 gestational weeks. The mean birth weight of the babies was 1,740.4±604.9 g (range 420 to 2,750 g). Group 1 included 7 boys and 13 girls with a mean birth weight of 2,085.0±391.3 g. Group 2 included 19 boys and 14 girls with a mean birth weight of 1,531.5±620.1 g. There were no significant differences between the two groups with respect to gender, type of delivery, gestational week, and birth weight.

**Table 1:** Demographic characteristics of the healthy (group 1) and unhealthy (group 2) preterm babies

		Group 1 (n=20)	Group 2 (n=33)	Total	p value
Gender [n (%)]	Male	7 (35%)	19 (57.6%)	26 (49.1%)	0.64
	Female	13 (65%)	14 (42.4%)	27 (50.9%)	0.83
Type of delivery [n (%)]	Vaginal delivery	2 (10%)	2 (6.1%)	4 (7.5%)	0.81
	Caesarean section	18 (90%)	31 (93.9%)	49 (92.5%)	0.93
Gestational weeks		34.2±1.8	30.9±3.3	32.1±3.2	0.16
Birth weight (g)		2,085.0±391.3	1,531.5±620.1	1,740.4±60.9	0.07

The presenting signs and symptoms in group 2 are shown in Table 2, the most frequent problems being RDS (87.9%) and sepsis (30.3%).

**Table 2:** The presenting signs and symptoms in group 2 (n=33)

	n	%
Respiratory distress syndrome	29	87.9
Sepsis	10	30.3
Bronchopulmonary dysplasia	4	12.1
Meconium aspiration syndrome	3	9.1
Pneumothorax	2	6.1
Asphyxia	2	6.1
Persistent pulmonary hypertension	1	3.0
Necrotizing enterocolitis	1	3.0
Surfactant therapy	29	87.9

The mean TSH levels of the two groups showed a consistent decline at three consequent measurements, which were always significantly lower in group 2 than in group 1 (for Day 1, p=0.004; for Day 7, p=0015; and for Day 14, p=0.049). In both groups, TSH levels showed significant decreases on Day 7 and Day 14 compared to the baseline levels (Table 3).

**Table 3:** Comparison of the TSH, FT_3_ and FT_4_ levels between the two groups

	TSH (uIU/ml)			FT_3_ (pg/ml)			FT_4_ (pg/ml)		
	Group 1 (Mean±SD)	Group 2 (Mean±SD)	^+^*p*	Group 1 (Mean±SD)	Group 2 (Mean±SD)	^+^*p*	Group 1 (Mean±SD)	Group 2 (Mean±SD)	^+^*p*
Day 1	15.1±13.9	8.0±8.3	*0.004*	3.1±1.3	2.3±0.6	*0.013*	1.5±0.4	1.3±0.5	*0.118*
Day 7	5.1±3.2	3.8±3.4	*0.015*	2.8±0.6	2.2±0.5	*0.001*	1.3±0.4	1.1±0.3	*0.055*
Day 14	3.0±1.5	2.4±1.2	*0.049*	2.6±0.6	2.2±0.6	*0.050*	1.3±0.3	1.1±0.3	*0.060*
^++^*p*	*0.001*	*0.001*		*0.056*	*0.823*		*0.102*	*0.062*	

^+^ Mann-Whitney U-test; ^++^ Friedman test.

The mean FT3 levels were significantly higher in group 1 than in group 2 at baseline and at 7 days, but this difference was not significant at 14 days (Table 3). Changes in FT3 levels in each group at 7 and 14 days showed no significant differences from the baseline levels. The mean FT4 levels were similar in the two groups at three consecutive measurements (Table 3).

The associations of TSH, FT3 and FT4 levels with gestational week and birth weight were as follows (Table 4): TSH showed no correlation with gestational week and birth weight at baseline (p>0.05); however, inverse correlations were noted with gestational week and birth weight at 7 and 14 days. On the other hand, FT3 and FT4 levels consistently showed significant correlations with gestational week and birth weight at each of the three measurements.

## Discussion

The most common thyroid function disorder in premature infants is physiological hypothyroxinemia, followed by hypothyroidism of non-thyroidal causes. In pre-maturity, several factors can inhibit the conversion of peripheral T4 to T3, including hypoxemia, acidosis, infections, hypoglycemia, hypocalcemia and malnutrition. Some rare conditions include transient secondary/tertiary hypothyroidism, transient primary hypothyroidism and permanent primary hypothyroidism.^10^ Patients with normal free T4 levels and transiently high TSH levels are considered to have transient elevation of TSH; low T3 levels in conjunction with low or normal free T4 and TSH levels are linked to non-thyroidal disease (euthyroid sick syndrome); a normal TSH level together with a transiently low free T4 level is called transient hypothyroxinemia, and a low free T4 level accompanied by a high TSH level denotes primary hypothyroidism.^5^,^6^,^10^ We found first day TSH levels high and FT4 levels normal in the healthy group and we considered this as transient TSH elevation. Except for this result, mean FT3 and FT4 levels were found normal in both groups.

A low FT4 level and a high TSH level have been reported to be risk factors for pre-term delivery, though no association has been shown between FT3 and pre-term delivery. 11,12 Infants born small for gestational age have also been shown to have higher TSH levels than those born appropriate for gestational age.^13^ In our study, while TSH showed no correlation with gestational week and birth weight at baseline (Day 1), it was inversely correlated with gestational week and birth weight at 7 and 14 days. FT3 and FT4 levels, on the other hand, consistently showed significant correlations with gestational week and birth weight at each of the three measurements.

In our study, TSH levels were higher in healthy pre-term infants than in unhealthy infants. Normal free T4 and transiently high TSH levels were considered transient elevation of TSH. The most frequent factors associated with neonatal transient hypothyroidism are maternal anti-thyroid drug use, maternal auto-antibodies, excess iodine intake and exposure, or iodine deficiency. Immaturity of the hypothalamic-pituitary-thyroid axis may account for the higher prevalence of transient hypothyroidism in premature infants than in term infants.^14^ In our study, exclusion criteria included maternal causes of thyroid disease, which are the most frequent causes of transient hypothyroidism, including maternal history of thyroid disease and abnormal thyroid function tests during pregnancy. However, this did not ensure elimination of other causes associated with transient hypothyroidism. Emphasis was placed rather on the relationship between thyroid function tests and complications of premature newborns. Thyroid dysfunction is common in infants admitted to intensive care units, most of which turn out to be euthyroid sick syndrome. Abnormal findings are often associated with transient increases in TSH during the recovery period of the condition. Therefore, close monitoring without further treatment usually suffices except in cases in which the TSH level is 15–20 mIU / L or remains high for longer than a month.^15^ Larson et al. reported that very low-birth-weight babies and infants receiving intensive care due to a body weight of less than 2500 g were at an increased risk for transient hypothyroidism and that use of dopamine and iodine resulted in delays in TSH elevation.^16^ In another study, infants with RDS had significantly lower levels of T4, FT4 and T3 on day 5 as compared with healthy pre-term infants but had similar TSH levels, suggesting that decreased thyroid function might be associated with RDS.^17^ In support of this finding, FT3 values were significantly lower at 1 and 7 days in group 2, of which 28/33 pre-term babies had RDS. Chen et al. found significantly lower levels of FT3 and FT4 in unhealthy pre-term babies as compared with healthy preterm babies.^18^ Chung et al. also found significantly lower FT4 levels in babies born before 28 weeks of gestation.^19^ They noted that low FT4 levels sustained during the first postnatal week but reached normal levels of term infants within the first two months. In addition, RDS and BPD were significantly more common in these infants than in the control group. In our study, serum FT4 levels were always lower in group 2 than in group 1, but the differences found at three measurements did not reach significance. Overall, in the setting of prematurity, TSH, FT3 and FT4 levels were lower in unhealthy infants than in healthy infants.

Thyroid dysfunction has been reported to be more common in infants with low birth weight due to inadequate intrauterine nutrition, hypoxia, and acidosis.^20^ In addition, a meta-analysis found that birth weight and gestation week were closely related to thyroid function tests.^21^ We also found significant correlations between thyroid function tests and birth weight and gestational age, which indicated increased thyroid dysfunction with lower birth weight and gestational age.

Our study has a number of important limitations. Higher degrees of iodine exposure is among the reasons for transient hypothyroisim in prematüre babies.^22^ The babies in our study were exposed to iodine containing antiseptics because of the procedures such as catheterization, blood collection and lumbar puncture in the intensive care unit. Pre-term infants in neonatal intensive care units are also subject to concomitant drugs, such as dopamine, metoclopramide, aminophylline and steroids to treat their illness, which result in transient thyroid dysfunction and affect test results.^5^ TSH and FT4 levels can vary significantly over the first 24 h and the first few days of life. Therefore, testing the hormone levels with routine morning blood sampling in the first 24 h of life is recommened.

## Conclusion

Pre-term infants, especially those having problems, have significant hypothyroxinemia during the first weeks of life. The majority of studies on thyroid hormone status in pre-term infants have been limited to the first week of life. We suggest that thyroid function tests should be repeated when found normal, especially in unhealthy prematures. Hypothyroxinemia represents an important risk factor for the development of pre-term infants, especially those having problems, and may require thyroid hormone replacement therapy for normal development and decreased morbidity. However, thyroid hormone replacement therapy has been assessed in a small number of studies, with varying combinations and doses and conflicting results. Even though we have not performed hormone replacement in our study, considering the importance of thyroid hormones in mental and motor development, replacement therapy may play an important role in reducing mortality and morbidity of premature infants as well as in improving their neuro-developmental processes throughout their follow-up. Further studies are warranted to substantiate these beneficial effects.
